# Increasing retirement ages in Denmark: Do changes in gender, education, employment status and health matter?

**DOI:** 10.1007/s10433-023-00771-0

**Published:** 2023-06-17

**Authors:** Anna Amilon, Mona Larsen

**Affiliations:** VIVE - The Danish Center for Social Science Research, Copenhagen, Denmark

**Keywords:** Retirement age, Cohort study, Blinder–Oaxaca decomposition, Education, employment status, Denmark

## Abstract

Recent studies report significant increases in retirement ages over the past two to three decades in most countries in the Organization for Economic Co-operation and Development—increases that research has attributed mainly to changes in the legislative frameworks for retirement in these countries. Using unique data from the Danish Longitudinal Study of Ageing, this study investigates whether and, if so to what extent, changes to the workforce in terms of gender, education, employment status (employed or self-employed) and health contribute to explaining differences in retirement ages between the cohorts born in 1935 and 1950. The retirement window of these cohorts stretches from the early 1990s to the late 2010s—a period characterized by substantial changes to workforce. On average, retirement ages increased by two years from the 1935 cohort to the 1950 cohort. However, due to changes in the investigated factors having offsetting effects, the net effect of such changes on retirement ages was minor. Thus, while increasing levels of education and better health among older workers contributed to increasing retirement ages, increasing female labour force participation and fewer self-employed workers had the opposite effect. In absolute terms, the total compositional and behavioural influence on retirement ages of changes in terms of employment status (− 0.35 years) was almost as large as the total changes in terms of education (0.44 years). Thus, future studies investigating long-term changes in retirement ages would benefit from including changes in employment status (self-employed or wage earner) as an explanatory factor.

**Supplementary Information:**

The online version contains supplementary material available at 10.1007/s10433-023-00771-0.

## Introduction

Population ageing throughout Western societies has raised concerns about increasing costs for old-age pensions and decreasing tax revenues (OECD 2019). In response, to boost labour force participation among older adults, many countries have increased statutory retirement ages and have restricted early-retirement opportunities (Mclaughlin and Neumark 2018). In addition to these changes in retirement legislation, a number of factors—including increasing educational levels, improved health among older workers and fewer physically demanding jobs (driven by technological change and a growing service sector)—may have contributed to the upward trend in retirement ages (OECD 2017a). Indeed, the last three decades have been characterized by increasing labour market participation rates among older adults in most countries in the Organization for Economic Co-operation and Development (OECD), including Denmark (OECD 2017b; Boissonneault et al. 2020; Bingley et al. 2021; Turek et al. 2022).

Research investigating the causes of the increases in retirement ages in OECD countries suggests that changes in retirement legislation have been an important driver (Boissonneault et al. 2020; Börsch-Supan and Coile 2018; Coile et al. 2018; Deeg et al. 2021; Geppert et al. 2019). Parallel to changes in retirement legislation, the gender, educational and occupational composition of the workforce has changed substantially (Albæk et al. 2022). The composition of the workforce is important for increasing retirement ages for the following two reasons. First, retirement ages are directly associated with workers’ characteristics, such as gender, education and health (Munnell 2015; OECD 2021). Second, changes in retirement behaviour (driven e.g. by legislative changes) may not be evenly distributed across socio-economic, demographic, educational and occupational groups (Coile et al. 2018; Radl 2013; Trentini 2020). For example, increases in retirement ages have mainly taken place among the highly educated (OECD 2017a; Rutledge 2018) and among people with high occupational status (Leinonen et al. 2020; Qvist 2020), and have in general been more pronounced for women than for men (OECD.Stat 2021).

While ample scholarly work has investigated causes of variation in exit ages both between individuals (Fisher et al. 2016; Scharn et al. 2018; Wang and Shultz 2010) and across countries (Börsch-Supan and Coile 2018; Larsen and Pedersen 2017), fewer studies have examined causes of variation in exit ages over time. Moreover, although research has established that changes in retirement legislation have constituted an important driver of increasing retirement ages (Börsch-Supan and Coile 2018; Geppert et al. 2019; Boissonneault et al. 2020), less is known about how retirement age is affected in the long term by the changes that have occurred in the old-age workforce.

This study investigates changes in retirement across cohorts (i.e. changes in retirement age over time). A recent systematic review of long-term changes in retirement ages (covering the period 2000–2019) identified 19 studies on this topic (Boissonneault et al. 2020). Among these studies, only three concerned demographic characteristics (including education). No study assessed the influence of increasing health or changes in employment status on retirement age.

This study thus contributes to the retirement literature by investigating whether and, if so to what extent, the increase in average retirement ages can be attributed to changes in the Danish workforce in terms of gender, education, employment status (shares of self-employed vs. wage earners) and health. We compare the cohorts born in 1935 and 1950, whose retirement window stretches from the early 1990s to late 2010s—a period characterized by substantial changes to retirement legislation as well as to the workforce.

In Denmark, changes in the retirement legislation primarily aimed at restricting early retirement opportunities (Larsen and Pedersen 2017). In particular, the *Voluntary Early Retirement Program* (VERP), which offered high replacement rates for low-wage workers, was subject to several reforms to reduce its attractiveness (Bingley et al. 2004). While the VERP initially aimed at offering early retirement to workers with physically demanding occupations, it gradually became a popular path to early retirement for broad segments of the population (Jørgensen 2009). To lower expenditures on the VERP, the statutory retirement age was actually *lowered* from 67 to 65 in 2004 (Larsen and Pedersen 2017). Thus, contrary to most OECD countries, the incentives for retiring after age 65 were attenuated from 2004 to 2019 in Denmark. Additional file 1: Online resource A provides a detailed overview of the legislative changes for the cohorts under study.

Changes to the workforce since the 1935 cohort entered the labour market (i.e. from the 1950s and onwards) include the outsourcing of labour-intensive production and technological development, leading to increased demand for highly skilled labour (Skaksen 2019) and to increases in average levels of education (Jacobsen 2004; Larsen et al. 2016). Consequently, since the 1950s, more highly educated white-collar workers have entered the Danish labour market while the share of unskilled blue-collar workers has decreased (Larsen and Pedersen 2017). At the same time, the gender distribution in the workforce has shifted as more women have entered the labour market (Statistics Denmark 2015) and the proportion of workers in good health has increased significantly (Siren and Larsen 2018).

This study is the first to investigate the potential impact of long-term changes in the share of self-employed workers in the workforce on the development of retirement ages. The share of self-employed workers has fallen dramatically in Denmark since the 1980s, and in 2019, only 6% of the workforce were self-employed, making it the lowest share in Europe. Potential explanations for this development include a shrinking agricultural sector and a growing public sector (Holstein 2021). While studies show a positive association between self-employment and retirement age at the individual level (Nolan and Barrett 2019; Anxo et al. 2019), the compositional and behavioural long-term influence of changes in self-employment on retirement ages has not previously been investigated. Given the dramatic fall in the share of self-employed workers, Denmark constitutes an important case for studying this association.

We use Blinder–Oaxaca decomposition to investigate changes in retirement ages across cohorts. This approach has the advantage of allowing for the decomposition of the increase in retirement age across cohorts into a “compositional”, a “behavioural” and an “interaction” component, where the first refers to changes in the characteristics of the workforce, the second refers to changes in retirement behaviour of the workforce and the third refers to interaction effects between compositional and behavioural changes (Jann 2008; Etezady et al. 2021). In other words, this analysis tells us to what extent the increase in retirement ages across cohorts are due to older workers being different (in terms of gender, education, employment status and health), acting differently or a combination of these factors.

## Expected impact of changes in characteristics

This study focuses on the cross-cohort impact of changes in terms of education, gender, employment status and health on increasing retirement ages. Drawing on retirement literature, we discuss the expected impact of each factor in the following paragraphs.

### Education

A number of studies have established a positive association between education and retirement age (e.g. Robroek et al. 2015; Turek et al. 2022). We therefore hypothesize a positive contribution to retirement ages from the increase in the shares of highly educated workers across cohorts (hypothesis H1a).

However, as relatively larger proportions of the workforce have obtained more education, the selection mechanisms into high-skilled occupations may have become softer. Changes in retirement legislation—which mainly aimed at limiting early retirement opportunities—may have amplified this potential gradual weakening of the skill-selection mechanism by boosting retirement ages among low-skilled workers (Bingley et al. 2019). We therefore expect the positive association between education and retirement age to have weakened over time (i.e. to be smaller for the 1950 than for the 1935 cohort) (hypothesis H1b).

Due to the offsetting influence of compositional and behavioural factors, we cannot form an a priori expectation as regard the total effect of changes in education on retirement ages.

### Gender

The increase in the share of female workers over time has likely influenced average retirement ages negatively, as women in general retire earlier than men (Axelrad and Mcnamara 2017; De Preter et al. 2013a, b) (hypothesis H2a). Moreover, weakening of the selection mechanism may have influenced the behaviour of female workers negatively, as employed women in the 1935 cohort were probably a more strongly positively selected group in terms of labour market attachment than employed women in the 1950 cohort. Hence, we expect the negative association between being female and retirement age to have increased over time (i.e. to be stronger for the 1950 than for the 1935 cohort) (hypothesis H2b). As we expect both compositional and behavioural effects in terms of gender to be negative, the total contribution of cross-cohort changes on retirement age is also expected to be negative (hypothesis H2c).

### Employment status

As self-employed workers generally retire later than wage earners (Nolan and Barrett 2019; Anxo et al. 2019), we expect the decrease in the share of self-employed workers across cohorts to have contributed negatively to retirement ages (hypothesis H3a). However, self-employed workers in the 1950 cohort were probably a more strongly selected group than self-employed workers in the 1935 cohort. If so, the positive association between self-employment and retirement age will be stronger for the 1950 than for the 1935 cohort (hypothesis H3b). Due to the offsetting influence of compositional and behavioural factors, we cannot form an a priori expectation as regard the total effect of changes in employment status on retirement ages.

### Health

Due to a positive association between good health and later retirement (De Preter et al. 2013a; Blundell et al. 2023), we expect the increasing shares of healthy workers across cohorts to have contributed positively to retirement ages (hypothesis H4a). Moreover, changes in retirement legislation have led to increasing incentives to prolong working lives—and workers in good health are more likely to have responded positively to such incentives (Mclaughlin and Neumark 2018). Therefore, we also expect an increasingly positive association between good health and retirement age across cohorts (hypotheses H4b). Consequently, the total effect of health and retirement age is also expected to be positive (hypothesis H4c).

The preceding paragraphs argue that the influence of changes across cohorts in terms of gender, education, employment status and health on retirement ages may be both positive and negative. Thus, the aggregated net effect of these changes on retirement ages remains an empirical question.

## Data and methods

### Sample

Our analysis builds on data from the Danish Longitudinal Study of Ageing (DLSA), a representative on-going longitudinal study of living conditions among older adults in Denmark, collected every five years since 1997 (Kjær et al. 2019). The data include several topics relevant to this study, including age at retirement, self-reported health and employment status during most of a person’s working life. The data include every fifth birth cohort from 1920 to 1960. In this study, we focus on individuals born in 1935 and 1950. We include individuals in the analysis who were in employment at age 50.

We mainly include survey data for these cohorts collected the year when respondents turned 67 years old, i.e. in 2002 and 2017. We choose to focus on data collected at age 67 for two reasons. First, most respondents have retired permanently from the labour market at age 67. Hence, by choosing this age, we can largely include information on actual (rather than expected or imputed) retirement age. Second, as most respondents retire in their 60 s, age 67 is, for most respondents, the earliest possible post-retirement data collection point.

However, retirement may have occurred substantially earlier (i.e. from age 50 and onwards), or may occur substantially later, than age 67. We therefore mainly include information on factors that are unlikely to change after age 50 in the analysis, including gender, education and employment status for most of one’s working life.

We combine the DLSA with information from the Danish administrative education registries, which include individual-level data on highest level of education for the entire population. Our final sample includes 2644 observations.

Our analysis compares the changes in characteristics and behaviour across the 1935- and the 1950-cohort. Thus, differences in attrition due to mortality or non-response across the two cohorts may influence results. However, 83% of 50-year-olds in the 1935-chort were still alive at age 67 whereas the corresponding figure in the 1950-cohort was 88% (Statistics Denmark 2023). Thus, differences in mortality across the two cohorts were minor.

Response rates in the DLSA have fallen over time and were 88% and 77% at age 67 in the 1935-and 1950-cohort, respectively. To investigate if this increasing attrition due to non-response may influence results, we compared the distribution of gender and education (available in registries) in the original sample and among respondents for the two cohorts. These analyses revealed no significant differences in the gender and educational distributions. While we cannot conduct a similar comparison for the survey-based variables, our comparison of key registry-based information suggests that our sample is representative for the general population for both cohorts.

### Variables

We use self-reported and imputed data to define our dependent variable, *age at retirement*. For those who had retired at or before age 67, we use the self-reported retirement age. For those retired people who did not report a retirement age, we impute retirement ages (restricted to the interval 50–67 years). Not all respondents had retired at age 67. Omitting these respondents would introduce bias, in particular as a larger proportion of respondents were still employed at age 67 in the 1950- than in the 1935-cohort (19.5 vs. 10.8%). For people in employment at age 67, we rely on expected retirement ages. While expected retirement ages overall have been found to be informative for actual retirement ages, there is a tendency towards respondents’ retiring earlier than expected (Kézdi and Shapiro 2023). However, we expect this tendency to be less pronounced in our data than in previous studies, as we only include expected retirement ages for those respondents who were still employed at age 67, whereas the literature on retirement expectations and realisations generally concerns workers that are younger than the statutory retirement age (see Kézdi and Shapiro (2023) and references therein). For those who did not report an expected retirement age, we impute retirement ages (restricted to the interval 67–80 years). In total, we impute retirement ages for 6% of the sample. A graph of the distribution of the dependent variable for the two cohorts appear in Additional file 1: Online resource B. While retirement to some degree is concentrated at pension accessibility ages, most people retire in their early- to mid-60 s, whereas few retire earlier or later. Thus, we argue that retirement ages are approximately normally distributed in both cohorts.

*Gender* is coded as either female or male.

The registry-based information on *education* is coded to form four levels: basic education, vocational education, short- or medium-cycle higher education (2–3 years), long-cycle higher education (at least 4 years).

The DLSA contains information on respondents’ employment status—e.g. whether respondents were white- or blue-collar workers or self-employed for most of their working lives. However, due to a strong correlation between skill-level (white- or blue-collar worker) and education, we define *employment status* based on self-reported information on whether respondents were self-employed or wage earners for most of their working lives.

Constructing a measure for *health* around the time of retirement is challenging, given that retirement ages have increased across cohorts (and health is correlated with age) and that the health care system has improved (making the usage of register-based health information problematic). We therefore use self-reported health at age 62 as a proxy for health around the time of retirement (using self-reported health at age 67 may introduce reversed causality, as most respondents have retired prior to that age). We impute health information for the 22% of the sample that were not interviewed at age 62 (or who had missing information on health). The health measure includes two levels: good (very good or good self-reported health) or bad (intermediate, bad or very bad self-reported health) (we do not use the full scale because few respondents reported bad or very bad health).

### Analytical approach

To investigate to what extent long-term changes to the workforce can explain increases in retirement ages we first descriptively analyse the development in workforce composition in terms of education, gender, employment status and health as well as changes in average retirement ages across cohorts in terms of these characteristics. Second, we use the Blinder–Oaxaca decomposition technique to examine if—and if so the extent to which—changes in gender, education, employment status and health across cohorts can explain the changes in retirement ages.

Following Jann (2008) and Etezady et al. (2021), we decompose the change in average retirement ages from the 1935 to the 1950 cohort into three terms, a “compositional”, a “behavioural” and an “interaction” component:

$$\begin{aligned}\Delta \overline{Y }&={\left({\overline{X} }_{1935}-{\overline{X} }_{1950}\right)}{^{\prime}}{\beta }_{1950}+{\overline{X} }_{1950}{^{\prime}}\left({\beta }_{1935}-{\beta }_{1950}\right)\\&\quad+{\left({\overline{X} }_{1935}-{\overline{X} }_{1950}\right)}{^{\prime}}\left({\beta }_{1935}-{\beta }_{1950}\right)\end{aligned}$$

The first term refers to changes in the characteristics of the workforce given retirement behaviour of the 1950 cohort (“compositional changes”), the second term refers to changes in the retirement behaviour given characteristics of the 1950 cohort (“behavioural changes”) and the third term refers to interaction effects between compositional and behavioural changes.

To avoid the choice of base category influencing behavioural results for categorical (dummy) variables, we express coefficients as deviations from the grand mean (Jann 2008).

### Handling missing data

We apply the Multiple Imputation of Chained Equations (MICE) procedure (Azur et al. 2011) to impute missing data on (a) retirement ages for retirees with missing information on this variable, (b) retirement ages for people in employment at age 67 with missing information on expected retirement age, and (c) self-reported health at age 62. We impute the missing values by regressing them one by one on information on gender, employment status (self-employed vs. wage earner), education, self-reported health at age 67 and year. Each cycle of one-by-one regressions on all other variables constitutes an iteration. By the end of the first iteration, the missing values on all the survey variables have been imputed. We perform 25 iterations, as previous research has shown that the number of imputations should be at least as large as the fraction of incomplete cases in the data (Bodner 2008; White et al. 2010). By 25 imputations, the data have sufficiently stabilized, such that the order in which variables are imputed no longer matters (Raghunathan et al. 2016). The imputed values after 25 iterations, combined with the observed data, constitute one imputed dataset. We repeat this procedure 25 times to generate 25 imputed datasets in total, and then use Rubin’s rule to combine the estimates from each of the 25 datasets to obtain the final results from the imputed data (Rubin 2004).

## Results

### Descriptive findings

Table 1 presents the distribution of the study sample on key variables by cohort. On average, retirement ages increased from 61.4 years in the 1935 cohort to 63.5 years in the 1950 cohort, i.e. by 2.1 years. The table clarifies substantial compositional differences across the cohorts in our study. While the share of female, healthy and highly educated workers increased, the share of self-employed and low-skilled workers decreased substantially. In particular, the share of self-employed workers fell by almost 50% from the 1935 to the 1955 cohort.

**Table 1:** Distribution on key variables by cohort

	1935 cohort	1950 cohort	Change 1950–1935
Retirement age (years) (Standard deviation)	61.4 (0.14)	63.5 (0.12)	2.08
Woman (%)	47.0	51.5	4.6
Self-employed (%)	18.8	9.9	− 9.0
Education (%):			
Basic education	47.2	22.1	− 25.0
Vocational education	32.5	41.4	8.9
Higher education (≤ 3 years)	16.1	29.2	13.1
Higher education (4 + years)	4.3	7.3	3.0
Good health at age 62 (%)	74.5	79.0	4.5
Observations	1035	1609	

**Fig. 1:**
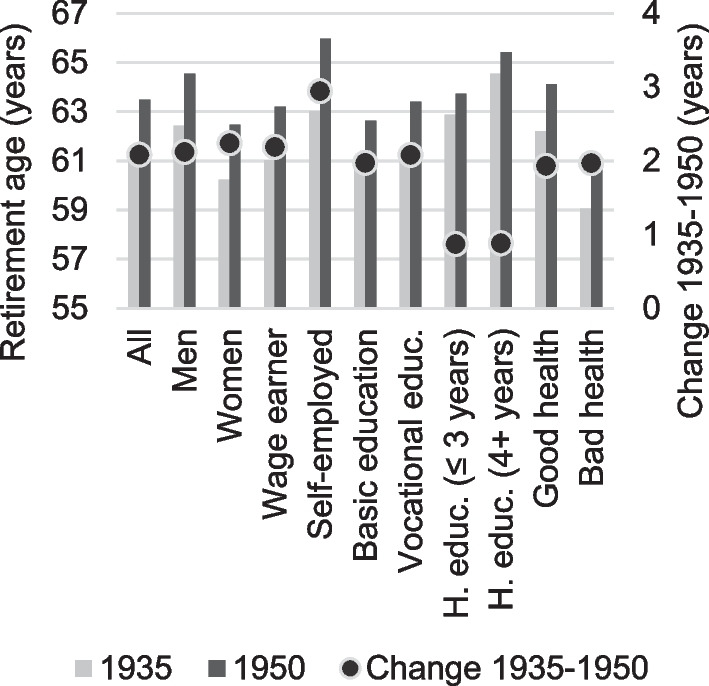
The development in average retirement ages across cohorts by socio-economic group (years)

Figure 1 presents average retirement ages by socio-demographic characteristics, as well as the change in average retirement ages from the 1935 to the 1950 cohort (secondary axis). While average retirement ages vary across socio-demographic groups, the change in retirement age across cohorts has, in general, been parallel between cohorts. For instance, while men retire approximately two years later than women do, this pattern has not changed across cohorts (average retirement ages have increased by approximately two years for both genders). We find the same parallel development for people in good vs. bad health. People in good health retire approximately three years later than people in bad health do, but regardless of health status, retirement ages grew by two years across the two cohorts.

However, we do not see this parallelism in terms of education and employment status. First, while there is an educational gradient in average retirement ages, the development across the two cohorts has been substantially slower among highly educated workers (approximately 0.9 years) than among workers with basic or vocational education (approximately 2 years). Changes in Danish retirement legislation aiming to reduce early retirement may explain these differences. Thus, in Denmark, in contrast to other high-income countries (OECD 2017a; Rutledge 2018; Bingley et al. 2019), workers with low levels of education have changed their retirement behaviour more than more highly educated workers.

Second, the self-employed workers stand out as the only group with higher-than-average retirement ages for both cohorts *and* a substantial (3 years) development across cohorts.

### Decomposition analysis

In Table 2, we study to what extent changes in terms of gender, education, employment status and health across cohorts can explain changes in retirement ages. Table 2 decomposes the increase in retirement ages from the 1935 cohort to the 1950 cohort into a compositional, a behavioural and an interaction component. The analysis also includes a constant term, which we assume mainly includes unobserved behavioural differences. In a decomposition analysis, the summation of all terms equals the total gap and therefore, a term cannot be set to zero due to low statistical significance (Etezady et al. 2021). Thus, standard errors are omitted from Table 2, but appear in Additional file 1: Online resource C.

**Table 2:** Decomposition analysis of increases in retirement ages from the 1935 cohort to the 1950 cohort. Changes in years in the composition-, behaviour- and interaction-term

	Composition	Behaviour	Interaction	Total
Average retirement age:				
1935 cohort: 61.40 years				
1950 cohort: 63.48 years				
Total change	0.21	2.05	− 0.18	2.08
Gender	− 0.08	0.00	0.00	− 0.08
Men	− 0.04	0.03	0.00	− 0.02
Women	− 0.04	− 0.02	0.00	− 0.06
Employment status	− 0.19	− 0.13	− 0.04	− 0.35
Wage earner	− 0.09	− 0.16	− 0.02	− 0.27
Self-employed	− 0.09	0.04	− 0.02	− 0.07
Education	0.35	0.23	− 0.14	0.44
Basic education	0.30	0.20	− 0.11	0.40
Vocational education	− 0.08	0.12	0.03	0.08
Higher education (≤ 3 years)	0.08	− 0.07	− 0.06	− 0.05
Higher education (4 + years)	0.04	− 0.01	− 0.01	0.02
Health	0.12	0.02	0.00	0.15
Good self-rated health	0.06	0.03	0.00	0.10
Bad self-rated health	0.06	− 0.01	0.00	0.05
Constant		1.91		
N				2644

Of the 2.1-year increase in retirement ages (from 61.4 years in the 1935 cohort to 63.5 years in the 1950 cohort), 0.21 years can be attributed to compositional changes. This rather limited influence is due to the changes in workforce composition offsetting each other. As hypothesized, relative increases in the shares of highly educated workers and workers in good health have influenced average retirement ages positively (by 0.35 + 0.12 = 0.47 years, or 5.6 months) (H1a, H4a), whereas changes in gender and employment status (i.e. relatively more female and fewer self-employed workers) have influenced retirement ages negatively (by − 0.08 − 0.19 = − 0.27 years, or − 3.2 months) (H2a, H3a).

The second column of Table 2 shows the impact of cross-cohort changes in behaviour on retirement age. Coefficients are expressed as deviations from the grand mean to avoid the choice of base category influencing behavioural results (Jann 2008). Therefore, the results related to behavioural changes tell us if the *difference* in behaviour within groups (e.g. the change in retirement ages that can be explained by differences in retirement behaviour between men and women) has increased or decreased across cohorts. However, the results do not tell us if that difference is mainly driven by a behavioural change, e.g. among men or among women. To clarify which groups mainly contribute to the observed changes in retirement behaviour, we present predicted retirement ages by characteristic and cohort in Additional file 1: Online resource D.

The estimates regarding behavioural changes, in-line with the descriptive findings, support the hypothesized patterns of a weakening association between educational level and late retirement (H1b), an intensifying negative association between being female and late retirement (H2b) and an intensifying positive association between being self-employed (H3b) and healthy (H4b) and late retirement.

The third column of Table 2 shows the contribution from the interaction between compositional and behavioural changes on retirement age. The total contribution from this term is negative and relatively small (− 0.18). The main part of this contribution comes from workers with basic education (− 0.14). This finding is due to the positive contribution from the relatively large increase in the retirement age for low-skilled workers largely being counterbalanced by this group becoming smaller over time.

Across compositional, behavioural and interaction effects, the total contribution of increasing levels of education is 0.44 years, whereas better health contributed positively by 0.15 years (H4c) to increasing retirement ages. The total contribution due to changes in the share of female workers (H2c) and self-employed workers both influenced retirement ages negatively, by − 0.08 and − 0.35 years, respectively. Thus, due to offsetting effects, changes across cohorts in gender, education, employment status and health explain only 0.16 years of the total 2.1-year increase in retirement ages. The increase in retirement ages is mainly due to behavioural changes that we cannot attribute to the investigated characteristics and that are captured by the constant term (1.91 years). Consequently, the increase in retirement ages from the 1935 to the 1950 cohort is mainly due to unobserved factors, including changes in retirement legislation (Boissonneault et al. 2020; Börsch-Supan and Coile 2018; Coile et al. 2018; OECD 2017b), that we cannot control for in our analysis.

## Discussion

This study analysed changes in retirement ages for the cohorts born in 1935 and 1950 across a retirement window stretching from the early 1990s to the late 2010s. Across cohorts, the composition of the labour force changed substantially, as increasing shares of highly educated and female workers entered the labour market, while the share of self-employed workers dropped considerably. While we recognize that changes in retirement legislation were important drivers of increasing retirement ages, this study contributes to the literature by examining the importance of changes in gender, education, employment status and health.

In particular, this study contributes by investigating the impact of changes in terms of employment status—i.e. changes in the shares and behaviour of wage earners and self-employed workers—on retirement ages. In total, the change in retirement ages attributed to changes in employment status amounts to − 0.35 years. Thus, changes in employment status across cohorts were, in absolute terms, more important for the development in retirement ages in Denmark than the corresponding changes in gender (− 0.08 years) and health (0.15 years) and almost as large as the changes in education (0.44 years). This study thus establishes the importance of including changes in employment status when explaining the long-term development in retirement ages.

While substantial changes to the workforce have taken place in the period examined here, these changes influence retirement ages both positively (e.g. increasing educational levels, and better health) and negatively (e.g. more female workers and fewer self-employed workers). Moreover, positive behavioural changes mainly took place among self-employed and low-skilled workers. However, these behavioural changes had little influence on aggregated retirement ages due to the negative compositional development (i.e. these groups becoming smaller over time). Thus, the net total effect of changes in gender, education, employment status and health on retirement ages has been limited.

In contrast to previous studies (OECD 2017a; Rutledge 2018), we find that increases in retirement ages have mainly taken place among low-skilled workers. In the retirement window under study, legislative changes in Denmark primarily aimed at restricting early retirement opportunities. Our study, in accordance with previous work, thus indicates that changes in retirement legislation are an important driver of increasing retirement ages (Boissonneault et al. 2020; Börsch-Supan and Coile 2018; Coile et al. 2018; OECD 2017b).

In addition, structural, demographical, technological and socio-cultural trends related to retirement behaviour may contribute to explaining increasing retirement ages across cohorts (de Wind et al. 2015; Browne et al. 2019). Fewer mentally and physically straining occupations, an increase in the demand for older workers due to demographic change and strong economic development during most of the retirement window under study are other factors that may have contributed to increasing retirement ages (DØRS 2021).

This study has three limitations that readers should consider when interpreting the findings. First, we only have access to expected or imputed retirement ages for people retiring past age 67, which prevents the precise estimation of the association between changes to the workforce and increases in retirement ages for this group. Second, we have limited information about respondents at the exact time of retirement. We have circumvented this problem by basing our analysis on variables that are unlikely to change as respondents’ approach retirement (education, gender and employment status for most of one’s working life). For health, we use health at age 62 as a proxy for health at the age of retirement. However, the precision of the proxy may vary across cohorts and hence, we may not accurately capture the true influence of changes in health on retirement ages. In addition, we cannot control for the labour force participation of respondents’ spouses, which has been found to influence retirement behaviour—in particular among men (Coile 2004, 2019; Schirle, 2008). Third, we cannot directly measure the influence of changes in the legislation regarding early retirement. Nevertheless, this study provides important and novel insights into the influence of compositional and behavioural changes—in particular in terms of employment status—on retirement ages.

## Conclusions

Over the last two to three decades, the demographic transition towards increasing shares of older adults has led to restrictions in retirement policies and increasing retirement ages across a wide range of countries (Bingley et al. 2019; Boissonneault et al. 2020; Turek et al. 2022). Parallel to legislative changes, significant changes to the workforce have occurred. Consequently, the workforce retiring in the early 1990s differed markedly from the workforce retiring in the late 2010s in terms of gender, education, employment status and health. This study analysed to what extent these changes to the workforce contribute to explaining the increase in retirement ages in Denmark over the past two to three decades. While our results show that changes in these factors had little *aggregated* impact on the increase in retirement ages, this limited impact was mainly due to them having offsetting effects. Therefore, our findings still have important implications for the future development in retirement ages—internationally as well as in Denmark.

For instance, the proportion of 60–70-year-olds with good health and educational resources can be expected to increase in the coming years, as the proportion of older people with physically demanding jobs decreases. Moreover, increasing numbers of workers have recently taken up self-employment following increasing unemployment rates due to the COVID-19 crisis (Statistics Denmark 2021; Nearside 2022; Utz et al. 2022). The demographic development and the associated labour shortages may contribute to continued high demand for older workers (OECD 2022). In addition, increasing retirement ages and other policy changes will likely influence retirement ages of future cohorts positively. All these factors point towards retirement ages that will increase in the future.

By contrast, increasing labour force participation among women is likely to put downward pressure on retirement ages in many countries. In addition, the “new” self-employed workers, who may have chosen self-employment to avoid COVID-19-related unemployment (i.e. out of necessity), may differ from previous generations of self-employed workers, who may have chosen self-employment more by desire. Thus, the association between being self-employed and late retirement might weaken in the future. Moreover, recent research demonstrate that not all workers are equally able to postpone retirement (Lain et al. 2019; Quinby and Wettstein 2021). In Denmark, this recognition has led to the introduction of a new early retirement program—“early pension”—that allows workers with a minimum work history of at least 42–44 years to retire one to three years before the statutory retirement age—and it is possible that other countries will follow suit. As the net-effect of these policy, compositional and structural changes is unknown, future research should continuously follow the development in retirement ages. Our results show that accounting for changes in employment status is an important explanatory factor when doing so.

## Supplementary Information

Below is the link to the electronic supplementary material.

**Additional file 1**. Supplementary file: Online resource (A–D).
