# Supplementary material for: Increasing retirement ages in Denmark: Do changes in gender, education, employment status and health matter?
Source: Eur J Ageing. 2023 Jun 17;20(1):24. doi: 10.1007/s10433-023-00771-0 (PMC10276799; doi:10.1007/s10433-023-00771-0)
Supplement: Supplementary file 1 — Additional file 1. Supplementary file: Online resource (A–D). [file 10433_2023_771_MOESM1_ESM.docx]

# SUPPLEMENTARY MATERIALS

# Online resource A - Retirement policies in Denmark

The Danish old-age pension is a three-pillar system, combining public Old Age Pension (OAP), and labor market and private pensions. The OAP is a pay-as-you-go, defined benefits plan whereas labor market and private pensions are privately funded, defined contribution plans. The OAP consists of two parts: a basic pension and a pension supplement. Both parts are means tested and payments are reduced if other incomes or earnings exceed certain thresholds. The pension supplement is tested against all sources of income (i.e., earnings, other pension income, interest rates etc.), whereas the basic pension is solely earnings tested.

In addition to the OAP, the cohorts studied in this paper had the option to advance their retirement via various early retirement schemes.

For instance, due to soaring unemployment rates, from 1992 until the beginning of 1996 it was possible for older unemployed workers to receive *transitional benefits* (80 per cent of unemployment benefits) through the Transitional Benefit Program (TBP). Thus, individuals born in 1935 could retire when they were between 56-59 years, conditional on having been unemployed in at least 12 out of the previous 15 months prior to their desired retirement date, and conditional on being a member of the *Voluntary Early Retirement Program* (VERP).

The VERP has allowed for non-health-related early retirement since 1979 (Bingley et al. 2021). Workers are eligible for VERP up to five years before the statutory retirement age, conditional on them having had a sufficiently long period of membership in an unemployment insurance fund and on them having been in the labor force until program entry (Larsen and Pedersen, 2017). While the VERP initially aimed at offering early retirement to workers with physically demanding occupations, it gradually grew increasingly popular, in particular among blue-collar workers. As the program’s main source of funding is general taxation and as VERP benefits are significantly higher than the OAP, successive Danish governments have, since the beginning of the 1990s, introduced several reforms to reduce the attractiveness of the VERP (Bingley *et al*., 2021; Larsen and Pedersen, 2013, 2017). In addition, in 2004, the government reduced the statutory retirement age from 67 to 65 to lower expenditures on the VERP.

In 1999, it became possible to work an unlimited number of hours (before 1999, there was a cap of 200 hours per year) while receiving the VERP (VERP benefits are reduced concurrently with increasing hours). Alternatively, by postponing program entry, individuals eligible for VERP could accumulate a tax rebate for each quarter they remained in the labor force until age 65 (Larsen and Pedersen, 2013). To further increase workers’ incentives to continue employment until the statutory retirement age (also for non-members of the VERP), those born in the period 1946-1952 who had been continuously employed full-time from age 60-64, and whose earnings while aged 57-59 did not exceed a certain threshold, received a tax rebate of up to DKK 100,000 at age 64 (ATP 2010) (exchange rate in 2022: GBP 1 ≈ DKK 8.9).

Prior to 2004, working past the statutory retirement age was highly unbeneficial due to means testing in the OAP. Therefore, the opportunity to *defer* OAP payments – and receive an actuarially fair compensation for deferring – was introduced in 2004, thereby substantially reducing the effective marginal tax rate caused by means testing (Amilon and Nielsen, 2010). In order to be eligible for deferred OAP, workers initially had to work at least 1,500 hours a year during the deferral period. This work requirement has gradually been reduced since its introduction and was 750 hours for the 1950 cohort.

Table A1 summarizes the main legislative changes that may have influenced the retirement behavior of individuals born in 1935 and 1950. Clearly, the incentives to postpone or advance retirement age in Denmark shifted across the two cohorts. Further, the impact of institutional changes may vary by, for example, gender and occupational group. For instance, changes to the VERP likely mainly affected women and low-skilled workers, who were the main participants in the VERP, whereas incentives to prolong working lives past the statutory retirement age mainly affected male, high-skilled workers.

In addition to the policy changes described in Table A1, the rules for Social Disability Pension (SDP) were restricted twice (in 2003 and 2013) in the retirement window in this study (Qvist 2020). However, as SDP can be granted at any age until the statutory retirement age, the influence of these restrictions by cohort are not clear cut. Nevertheless, the set of rules governing SDP were more lenient for the 1935 cohort than for the 1950 cohort.

Lastly, unemployment rates in Denmark fell from around 12 per cent in the early 1990s to 2 per cent in 2008 and while it increased in connection with the financial crisis, it has since remained low (at around 4 per cent) during the early-to-mid 2010s (Danmarks Statistik 2021). Thus, due to economic recovery and gradual population ageing, demand for old-age workers likely increased substantially in the retirement window studied here.

**Table A1.** Institutional framework regulating retirement options, by cohort.

|  | 1935 cohort  Retirement ages: 56-67+  (1991-2002+) | 1950 cohort  Retirement ages: 60-65+  (2010-2015+) |
| --- | --- | --- |
| **Transitional Benefit Program (TBP)** |  |  |
| Available | Yes | No |
| Age of availability | 56-59 years | - |
| **Voluntary Early Retirement Program (VERP)** |  |  |
| Earliest age of access | 60 years | 60 years |
| Incentives to postpone program entry | Yes, until age 63. | Yes, until age 62. |
| Work while on the program | 200 hours / year without reduction in benefits. | No work limit. Proportional reduction in benefits. |
| Reduced tax on earnings from age 62. | No | Yes |
| **Old age pension (OAP)** |  |  |
| Statutory retirement age | 67 | 65 |
| Reduced tax on earnings at age 64 | No | Yes, DKK 100000 |
| Reduced tax on earnings from age 65 | No | Yes, DKK 60000 |
| **Deferred OAP** |  |  |
| Available | No | Yes |
| Work requirement (hours / year) | - | 750 hours |

*Note:* “Retirement ages” refer to the earliest and latest age of accessibility to the policies described in the table. Years in parentheses refer to the retirement window of the cohort.

# Online resource B. Distribution of dependent variable (age at retirement) by cohort

**Fig. B1** Distribution of age at retirement by cohort

Notes: Retirement age is imputed for 6 percent of the sample (151 observations). The value of retirement age for these 151 observations is based on the mean value of the 25 imputations rounded to the closest integer.

We show retirement after age 70 in intervals due to few observations.

# Online resource C. Additional details of decomposition analysis

**Table C1.** Decomposition analysis of increases in retirement ages from the 1935 cohort to the 1950 cohort. Changes in years in the composition-, behavior- and interaction-term. Coefficients and Standard Errors.

|  | 1935-1950 | | |
| --- | --- | --- | --- |
|  | Coefficient | | SE |
|  | Overall | |  |
| 1950 cohort | 63.48 | *** | 0.11 |
| 1935 cohort | 61.40 | *** | 0.14 |
| Difference | 2.08 | *** | 0.18 |
| Composition | 0.21 | ** | 0.11 |
| Behavior | 2.05 | *** | 0.18 |
| Interaction | -0.18 | * | 0.10 |
|  | Composition^a^ | |  |
| Gender (total) | -0.08 | ** | 0.04 |
| Occupation (total) | -0.19 | *** | 0.04 |
| Education (total) | 0.35 | *** | 0.08 |
| Unskilled | 0.30 | *** | 0.06 |
| Vocational | -0.08 | ** | 0.03 |
| Higher education (≤ 3 years) | 0.08 | ** | 0.04 |
| Higher education (4+ years) | 0.04 | ** | 0.02 |
| Health (total) | 0.12 | ** | 0.05 |
|  | Behaviour | |  |
| Gender (total) | 0.00 |  | 0.09 |
| Men | 0.03 |  | 0.08 |
| Women | -0.02 |  | 0.08 |
| Occupation (total) | -0.13 |  | 0.15 |
| Wage earner | -0.16 |  | 0.20 |
| Self-employed | 0.04 |  | 0.05 |
| Education (total) | 0.23 |  | 0.17 |
| Basic education | 0.20 |  | 0.15 |
| Vocational education | 0.12 |  | 0.10 |
| Higher education (≤ 3 years) | -0.07 |  | 0.06 |
| Higher education (4+ years) | -0.02 |  | 0.02 |
| Health (total) | 0.02 |  | 0.10 |
| Good health at age 62 | 0.03 |  | 0.16 |
| Bad or intermediate health at age 62 | -0.01 |  | 0.05 |
| Constant | 1.91 | *** | 0.32 |
|  | Interaction^a^ |  |  |
| Gender (total) | -0.00 |  | 0.01 |
| Occupation (total) | -0.04 |  | 0.04 |
| Education (total) | -0.14 |  | 0.10 |
| Basic education | -0.11 |  | 0.08 |
| Vocational education | 0.03 |  | 0.03 |
| Higher education (≤ 3 years) | -0.06 |  | 0.05 |
| Higher education (4+ years) | -0.01 |  | 0.02 |
| Health (total) | 0.00 |  | 0.02 |
|  | Unobserved | |  |
| Observations | 2644 |  |  |

*Notes:* SE: standard error. * *p* < 0.10, ** *p* < 0.05, *** *p* < 0.01. ^a^ For binary variables, a compositional increase in one factor is per definition accompanied by a corresponding decrease in the other. Therefore, we only present the total effect of binary variables in the “Composition”- and “Interaction”-section of our analysis.

# Online resource D

**Table D1.** Adjusted linear predictions of retirement ages by socio-economic characteristic and cohort.

|  | 1935 | |  | 1950 | |  | 1950-1935 |
| --- | --- | --- | --- | --- | --- | --- | --- |
|  | Predicted retirement age | SE |  | Predicted retirement age | SE |  |  |
| Men | 62.38 | 0.18 |  | 64.36 | 0.15 |  | 1.99 |
| Women | 60.67 | 0.20 |  | 62.56 | 0.15 |  | 1.89 |
|  |  |  |  |  |  |  |  |
| Wage earner | 61.25 | 0.15 |  | 63.13 | 0.11 |  | 1.88 |
| Self-employed | 63.32 | 0.32 |  | 65.60 | 0.34 |  | 2.29 |
|  |  |  |  |  |  |  |  |
| Basic education | 60.79 | 0.19 |  | 63.00 | 0.22 |  | 2.21 |
| Vocational education | 61.15 | 0.23 |  | 63.31 | 0.16 |  | 2.16 |
| Higher educ (≤ 3 years) | 62.62 | 0.32 |  | 63.97 | 0.20 |  | 1.35 |
| Higher educ (4+ years) | 63.43 | 0.64 |  | 64.87 | 0.39 |  | 1.44 |
|  |  |  |  |  |  |  |  |
| Good self-rated health | 62.15 | 0.16 |  | 64.11 | 0.12 |  | 1.96 |
| Bad self-rated health | 59.39 | 0.29 |  | 61.25 | 0.24 |  | 1.87 |

The predicted retirement ages are based on linear regression models with interaction terms between all explanatory variables and cohort. Predictions are estimated at the means of the covariates. SE = Standard Error.

# References to supplementary materials

Amilon A, Nielsen TH (2010) How does the option to defer pension payments affect the labour supply of older workers in Denmark? In: van Loo J, Bohlinger S (eds) Working and ageing - Emerging theories and empirical perspectives. Publications Office at the European Union, Luxembourg

ATP (2010) Skatterabat på op mod 60.000 kr. på vej til ældre. faktum

Bingley P, Datta Gupta N, Kallestrup-Lamb M, Pedersen PJ (2021) Labor Force Exit in Denmark 1980-2016: Impact from Changes in Incentives. University of Chicago Press

Danmarks Statistik (2021) AULAAR: Fuldtidsledige (netto) efter køn og personer/pct. In: Statistikbanken. https://www.statistikbanken.dk/statbank5a/default.asp?w=1280. Accessed 15 Jun 2021

Larsen M, Pedersen PJ (2017) Labour force activity after 65: what explain recent trends in Denmark, Germany and Sweden? J Labour Mark Res 50:15–27. https://doi.org/10.1007/s12651-017-0223-7

Larsen M, Pedersen PJ (2013) To work, to retire – or both? Labor market activity after 60. IZA J Eur Labor Stud 2:. https://doi.org/10.1186/2193-9012-2-21

Qvist JY (2020) The working class and early retirement in Denmark: Individual push factors. Ageing Soc. https://doi.org/10.1017/S0144686X20000203
